# Cytoplasmic Viral RNA-Dependent RNA Polymerase Disrupts the Intracellular Splicing Machinery by Entering the Nucleus and Interfering with Prp8

**DOI:** 10.1371/journal.ppat.1004199

**Published:** 2014-06-26

**Authors:** Yen-Chin Liu, Rei-Lin Kuo, Jing-Yi Lin, Peng-Nien Huang, Yi Huang, Hsuan Liu, Jamine J. Arnold, Shu-Jen Chen, Robert Yung-Liang Wang, Craig E. Cameron, Shin-Ru Shih

**Affiliations:** 1 Research Center for Emerging Viral Infections, College of Medicine, Chang Gung University, Tao-Yuan, Taiwan; 2 Graduate Institute of Biomedical Sciences, College of Medicine, Chang Gung University, Tao-Yuan, Taiwan; 3 Department of Medical Biotechnology and Laboratory Science, College of Medicine, Chang Gung University, Tao-Yuan, Taiwan; 4 School of Medical Laboratory Science and Biotechnology, Taipei Medical University, Taipei, Taiwan; 5 Molecular Medicine Research Center, Chang Gung University, Tao-Yuan, Taiwan; 6 Department of Biochemistry and Molecular Biology, Pennsylvania State University, University Park, Pennsylvania, United States of America; 7 Department of Biomedical Sciences and Graduate Institutes of Biomedical Sciences, College of Medicine, Chang Gung University, Tao-Yuan, Taiwan; 8 Clinical Virology Laboratory, Chang Gung Memorial Hospital, Tao-Yuan, Taiwan; University of North Carolina at Chapel Hill, United States of America

## Abstract

The primary role of cytoplasmic viral RNA-dependent RNA polymerase (RdRp) is viral genome replication in the cellular cytoplasm. However, picornaviral RdRp denoted 3D polymerase (3D^pol^) also enters the host nucleus, where its function remains unclear. In this study, we describe a novel mechanism of viral attack in which 3D^pol^ enters the nucleus through the nuclear localization signal (NLS) and targets the pre-mRNA processing factor 8 (Prp8) to block pre-mRNA splicing and mRNA synthesis. The fingers domain of 3D^pol^ associates with the C-terminal region of Prp8, which contains the Jab1/MPN domain, and interferes in the second catalytic step, resulting in the accumulation of the lariat form of the splicing intermediate. Endogenous pre-mRNAs trapped by the Prp8-3D^pol^ complex in enterovirus-infected cells were identified and classed into groups associated with cell growth, proliferation, and differentiation. Our results suggest that picornaviral RdRp disrupts pre-mRNA splicing processes, that differs from viral protease shutting off cellular transcription and translation which contributes to the pathogenesis of viral infection.

## Author Summary

RNA-dependent RNA polymerase (RdRp) is an enzyme that catalyzes the replication from an RNA template and is encoded in the genomes of all RNA viruses. RNA viruses in general replicate in cytoplasm and interfere host cellular gene expression by utilizing proteolytic destruction of cellular targets as the primary mechanism. However, several cytoplasmic RNA viral proteins have been found in the nucleus. What do they do in the nucleus? This study utilized picornaviral polymerase to probe the function of RdRp in the nucleus. Our findings reveal a novel mechanism of viruses attacking hosts whereby picornaviral 3D polymerase (3D^pol^) enters the nucleus and targets the central pre-mRNA processing factor 8 (Prp8) to block pre-mRNA splicing and mRNA synthesis. The 3D^pol^ inhibits the second catalytic step of the splicing process, resulting in the accumulation of the lariat-form and the reduction of the mRNA. These results provide new insights into the strategy of a cytoplasmic RNA virus attacking host cell, that differs from viral shutting off cellular transcription and translation which contributes to the viral pathogenesis. To our knowledge, this study shows for the first time that a cytoplasmic RNA virus uses its polymerase to alter cellular gene expression by hijacking the splicing machinery.

## Introduction

RNA viruses in general replicate in the cytoplasm and interfere host cellular gene expression by utilizing proteolytic destruction of cellular targets as the primary mechanism [1]. However, several viral proteins have been found in the nucleus and altered host gene expression [2]. For example, our previous finding shows that picornaviral 3C protease cleaves CstF-64 and inhibits cellular polyadenylation in the nucleus [3]. Besides the protease, the RNA-dependent RNA polymerase (RdRp) also appears in the nucleus, but the role of viral RNA polymerase in the nucleus remains unclear. This study utilized picornaviral polymerase to probe the function of RdRp in the nucleus.

Picornaviruses cause numerous diseases in humans and various animal species. The enteroviruses in the *Picornaviridae* family are critical human pathogens that typically cause hand, foot, and mouth disease (HFMD) and contribute to severe neurological complications, including aseptic meningitis, brainstem encephalitis, poliomyelitis, and even death [4], [5]. Enterovirus 71 (EV71) has played an increasingly substantial role in emerging epidemics around the Asia Pacific region, and these infections are particularly life-threatening in young children [6]–[8].

Picornaviruses have a single-stranded, positive-sense RNA genome. This genome encodes the RdRp, also known as 3D polymerase (3D^pol^), which becomes active upon completion of the auto-catalyzed proteolytic processing of the protease-polymerase precursor 3CD [9], [10]. 3D^pol^ plays a central role in viral genome replication in the cytoplasm of infected cells by catalyzing the uridylylation of small protein VPg (VPg-pUpU) as a primer during viral RNA replication [11]–[13]. The cellular phosphatidylinositol-4-phosphate (PI4P) lipid-enriched microenvironment is essential for viral RNA replication, and the 3D^pol^ specifically binds PI4P to catalyze the synthesis of viral RNA [14]. The 3D^pol^ of picornaviruses exhibit a similar overall structure, which can be described as a right hand with fingers, palm, and thumb domains. The fingers domain creates the entrance to facilitate the entry and stabilization of the template RNAs. In addition to the 3 central domains, an N-terminal domain that bridges the fingers and thumb domains is observed in all RdRps. The amino acid sequence and the structural elements of 3D^pol^ are conserved in evolutionarily distant species and serve essential functional roles [15], [16]. 3D^pol^ is a potential target for drug discovery based on its critical role in viral replication and its structure and sequence conservation [16]–[18].

Although picornaviral 3D^pol^ primarily performs viral replication in the host cytoplasm, 3CD and 3D^pol^ are capable of entering the nucleus in virus-infected cells [2], [19]–[22]. 3CD and 3D^pol^ of poliovirus (PV) enter the nucleus through a nuclear localization signal (NLS), KKKRD, which spans 125–129 amino acids (aa) within 3D^pol^. The putative NLS in the 3D^pol^ coding region is partially contained within the sequence KKRD (126–129 aa), which is typical among all known picornaviral 3D^pol^ [2], [22]. Picornaviral 3C is delivered into the nucleus through its precursor 3CD, which results in the cleavage of cellular transcriptional factors or regulators, such as CstF-64, polymerase I factor SL-1, TATA-box binding protein (TBP), cyclic AMP-responsive element binding protein (CREB), Octamer binding protein-1 (Oct-1), p53, histone H3, and RNA polymerase III transcription factor IIIC [2], [3], [23]–[28]. However, previous studies have only demonstrated that 3D^pol^ containing a NLS can transport 3CD to the nucleus, and the precise role of 3D^pol^ in the host cell nucleus remains unclear. In this study, we identified several nuclear target proteins in U5 small nuclear ribonucleoprotein particles (U5 snRNPs) that interact with 3D^pol^.

U1, U2, U4, U5, and U6 snRNPs are essential components of the spliceosome in the pre-mRNA splicing process, which involves introns excision and exon ligation to form a mature mRNA. U5 snRNPs contain several functionalities crucial to pre-mRNA splicing factors, such as Prp8/220K, Brr2/200K, Snu114/116K, Prp6/102K, Prp28/100K, Lin1/52K, SNRNP40/40K, and Dib1/15K [29]–[31]. The human pre-mRNA processing factor 8 (Prp8), one of the largest and highly-conserved nuclear proteins, provides a large scaffold and occupies a central position in the catalytic core of a spliceosome. Prp8 contains 5 functional domains: NLS, RNA recognition motif (RRM), 3′ splice site (3′SS) fidelity region, 5′ splice site (5′SS) holding domain, and Jab1/MPN. The putative bipartite NLS of Prp8 enables Prp8 to enter the nucleus, assisted by the import-α family, and the RRM domain provides an RNA binding center for pre-mRNA. The 5′SS holding domain of Prp8 is believed to lock the 5′SS-OH (exon) and load it onto the 3′SS-associated region of Prp8. The Jab1/MPN domain in Prp8 represents a pseudo-enzyme that is converted into a protein-protein interaction platform. Prp8 is the only spliceosomal protein that directly cross-links to the 5′SS, 3′SS, and branch point (BP) of the pre-mRNA substrate, as well as to U2, U5 and U6 snRNAs. Prp8 also interacts with several spliceosomal proteins, including the RNA helicase Brr2, the GTPase Snu114, and Prp6 of the U5 snRNP. The human Prp8 terminal fragment, the N-terminal region containing the NLS domain and the C-terminal region containing the Jab1/MPN domain also interact with each other, suggesting that these regions form an intermolecular bridge [32]–[38].

In pre-mRNA processing, the U1 snRNP initially binds to the 5′SS, and the U2 snRNP associates with the BP of the pre-mRNA. The U5•U4/U6 tri-snRNPs assembly then forms the pre-spliceosome B-complex. Then, a major structural change occurs upon the release of the U1 and U4 snRNPs, transforming the B-complex into the catalytically active component (B^act^-complex) of the spliceosome. The first catalytic step of the splicing process involves the B^act^-complex catalyzing the first *trans*-esterification reaction, which is the attack of the branch site 2′-OH on the 5′SS. This produces exon1 and the lariat form (intron-exon2), which forms the C1-complex. The second catalytic step is to transition from the C1 to C2 complex for the second *trans*-esterification. The attack of the 3′-OH of the 5′exon on the 3′SS results in 3′SS cleavage and exon ligation to produce the mRNA and release the excised intron [39]–[41]. Prp8, U5 snRNA, and the nonspliceosomal proteins Prp16, Slu7, Prp18, and Prp22 are required for the second catalytic step. In addition, Arg1753 of Prp8 cooperates with Prp18 to stabilize the U5/exon contacts that are crucial for the second catalytic step and ensure that the Prp22 helicase disrupts the interactions between the U5 snRNP and mRNA to release the mRNA [36], [42].

In this study, we describe a novel mechanism for picornavirus invasion of host cells that involves a previously unidentified function of 3D^pol^ that differs from its classic role in viral replication. Our results suggest that the 3D^pol^ enters the cellular nucleus to associate with the core splicing factor Prp8. Furthermore, 3D^pol^ affects the normal function of Prp8 during the second catalytic splicing step, leading to the inhibition of pre-mRNA splicing, the accumulation of the lariat form, and a decrease in the resulting mRNA. This is the first study demonstrating that a cytoplasmic RNA virus can use its polymerase to alter cellular gene expression by hijacking the splicing machinery.

## Results

### 3D^pol^ specifically interacts with the nuclear protein Prp8

**Figure 1:**
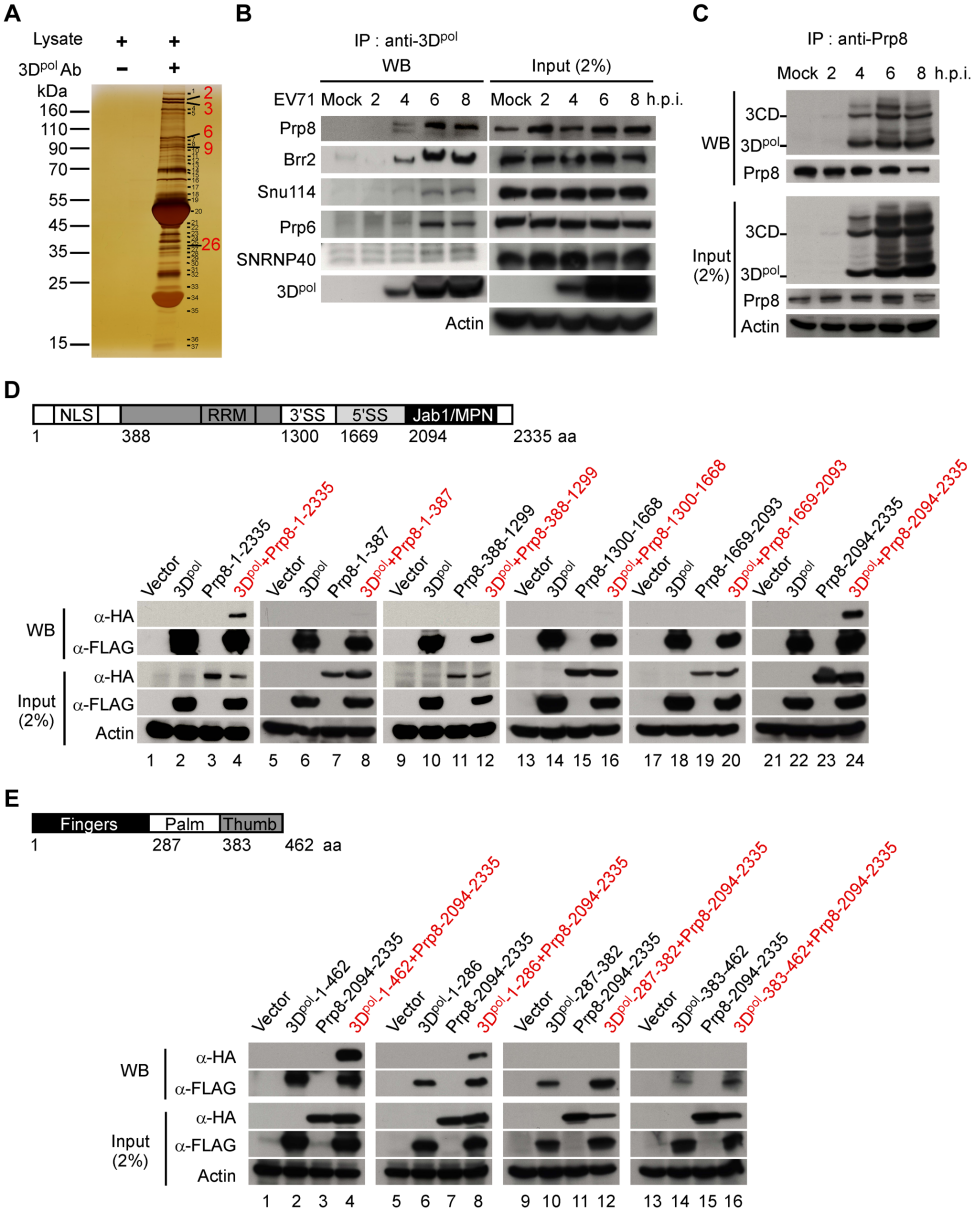
3D^pol^ associates with the nuclear protein Prp8. (A) Identification of potential 3D^pol^-interacting host proteins. The cell lysates for IP were harvested from EV71 40 MOI-infected RD cells at 6 h.p.i. and treated with the 3D^pol^ monoclonal antibody or untreated as a negative control. The proteins that interacted with 3D^pol^ were pulled down using an anti-3D^pol^ antibody, along with protein A-Sepharose, and detected by 1D SDS-PAGE and silver staining. (B) 3D^pol^ interacts with 5 components of U5 snRNPs, including Prp8, Brr2, Snu114, Prp6, and SNRNP40. The interaction of EV71 3D^pol^ and the nuclear protein U5 snRNPs was further confirmed by Co-IP and WB assays. The lysates harvested from mock- or EV71 40 MOI-infected RD cells at 2 to 8 h.p.i. were treated with RNase A (10 µg/ml) and immunoprecipitated using an anti-3D^pol^ antibody. The 5 components of the U5 snRNPs that interacted with 3D^pol^ were detected using a WB assay. The input samples were verified in the presence of 3D^pol^ and the five components of the U5 snRNPs in the lysates. Actin served as an internal control. (C) The core spliceosome splicing factor Prp8 can also pull down 3D^pol^ and 3CD. EV71-infected RD cell lysates from 2 to 8 h.p.i. were treated with RNase A (10 µg/ml) and incubated with antibodies against the Prp8 probe. After the IP assay, 3D^pol^ and 3CD were analyzed using WB with an anti-3D^pol^ antibody. (D) 3D^pol^ associates with the C-terminal domain of Prp8 containing the Jab1/MPN region. The functional domain architecture of human Prp8 is shown (upper panel). HEK293T cells were transfected with plasmids encoding full-length FLAG-3D^pol^ (lanes 2, 4, 6, 8, 10, 12, 14, 16, 18, 20, 22, and 24), various truncated forms of HA-Prp8 (lanes 3, 4, 7, 8, 11, 12, 15, 16, 19, 20, 23, and 24), and empty vectors (lanes 1, 5, 9, 13, 17, and 21). At 48 h after transfection, the lysates were treated with RNase A (10 µg/ml) and immunoprecipitated with antibodies against FLAG. The truncated form of Prp8 that interacted with 3D^pol^ was detected by WB using an antibody against HA. (E) The C-terminal domain of Prp8 interacts with the fingers domain of 3D^pol^. The functional domain architecture of EV71 3D^pol^ is shown (upper panel). HEK293T cells were transfected with plasmids encoding HA-Prp8-2094-2335 (lanes 3, 4, 7, 8, 11, 12, 15, and 16), various truncated forms of FLAG-3D^pol^ (lanes 2, 4, 6, 8, 10, 12, 14, and 16), and empty vectors (lanes 1, 5, 9, and 13). The various truncated forms of 3D^pol^ were pulled down by IP with an anti-FLAG antibody. The C-terminal domain containing the Jab1/MPN region of Prp8, which interacts with the truncated form of FLAG-3D^pol^, was detected with an anti-HA antibody in a WB assay.

Picornaviral 3D^pol^ has been shown to enter the nucleus of infected cells [2], [20], [22]; however, the role of 3D^pol^ in the nucleus has not been explored. We generated an EV71 3D^pol^ monoclonal antibody that could recognize 3D^pol^ in the lysates of RD cells infected with EV71 at a multiplicity of infection (MOI) of 40. To determine the role of 3D^pol^ in the host nucleus, the 3D^pol^-interacting proteins were pulled down and detected using a one-dimensional sodium dodecylsulfate-polyacrylamide gel electrophoresis (1D SDS-PAGE) assay (Figure 1A). These potential target proteins were identified using matrix-assisted laser desorption ionization-time of flight mass spectrometry (MALDI-TOF MS) analysis, and the results are summarized in Table 1. These data indicated that 3D^pol^ may interact with numerous U5 snRNP nuclear proteins, including Prp8, Brr2, Snu114, Prp6, and SNRNP40. This interaction was further confirmed in EV71-infected RD cell lysates following RNase A treatment using co-immunoprecipitation (Co-IP) and Western blotting (WB) assays (Figure 1B). Prp8, which is the nuclear protein at the central position in the catalytic core of the spliceosome, was selected for further study. The interaction of Prp8 and 3D^pol^ was further verified in EV71-infected RD cell lysates following RNase A treatment by Co-IP and WB assays with antibodies against endogenous Prp8 and viral 3D^pol^, respectively. The result of these assays suggested that endogenous Prp8 interacts with EV71 3D^pol^ and 3CD between 4 to 8 h post-infection (h.p.i.), without intermediation of the RNA (Figure 1C). To discover the interacting domains of Prp8 and 3D^pol^, we constructed tags that were fused with hemagglutinin (HA) or FLAG epitopes for the various truncated forms of Prp8 and 3D^pol^, respectively, and the various fragments of Prp8 and 3D^pol^ were cloned separately from each functional domain. To identify which Prp8 domain is responsible for the 3D^pol^ interaction, plasmids containing various truncated forms of Prp8 fused with HA and the FLAG-tagged full-length 3D^pol^ were transfected into HEK293T cells, followed by anti-FLAG IP and WB assays. The results of these assays revealed that the full-length Prp8 (lane 4) and the C-terminal region (2094–2335 aa) containing the Jab1/MPN domain of Prp8 (lane 24) interacted with full-length 3D^pol^ (Figure 1D). Furthermore, to determine which domain of 3D^pol^ interacts with the C-terminal region of Prp8, we used different FLAG-tagged truncated forms containing the fingers, palm, and thumb domains, as well the HA-tagged C-terminal region, in anti-FLAG IP and WB assays. This mapping study revealed that the fingers domain in the N-terminal region (1–286 aa) of 3D^pol^ interacts with the C-terminal region (2094–2335 aa) of Prp8 (lane 8) (Figure 1E). Here, we demonstrate that 3D^pol^ associates with the nuclear protein Prp8 via a protein-protein interaction that involves at least one region between the finger domain of 3D^pol^ and the C-terminal domain of Prp8.

**Table 1:** Potential protein targets of EV71 3D^pol^ were identified by MALDI-TOF MS analysis.

Band Number	Protein Name	NCBI GI No.	Score	Sequence Coverage (%)	Mass (Da)
2	Pre-mRNA processing factor 8 homolog (Prp8) (220 kDa U5 snRNP-specific protein)	gi|39963074	339	38	274719
3	200 kDa U5 snRNP-specific spliceosomal protein (Brr2)	gi|45861372	391	44	246032
6	U5-116KD (Snu114)	gi|48145665	223	37	110360
9	U5 snRNP associated 102 kDa protein (Prp6)	gi|119595584	146	39	101447
10	Aminopeptidase puromycin sensitive	gi|119615217	72	27	93428
14	Sec23a24A HETERODIMER, Complexed With The Snare Protein Sec22b	gi|149242495	75	30	87320
15	78 kDa glucose-regulated protein	gi|16507237	60	35	72402
16	Heat shock 70 kDa protein 8 isoform 1	gi|5729877	136	52	71082
17	Ras-GTPase-activating protein SH3-domain-binding protein	gi|119582066	91	48	56658
23	Selenium donor protein	gi|1000284	75	26	42754
24	Eukaryotic translation initiation factor 3, subunit 3 gamma	gi|4503515	88	53	40076
26	U5 snRNP-specific 40 kDa protein (SNRNP40)	gi|3820594	115	54	39730
27	Eukaryotic translation initiation factor 3, subunit 2 beta	gi|4503513	86	55	36878
32	40S ribosomal protein S3	gi|15718687	59	48	26842
33	Heat shock protein beta-1	gi|4504517	60	37	22826

Footnotes:

The score of the above-mentioned proteins was greater than 50. The band numbered 1, 4–5, 7–8, 11–13, 18–22, 25, 28–31, 34–37 did not achieve a significant score for protein identification and were not listed in the Table.

The sequence coverage for these proteins determined by MALDI-TOF analysis is also indicated.

### 3D^pol^ enters the cellular nucleus and colocalizes with Prp8

**Figure 2:**
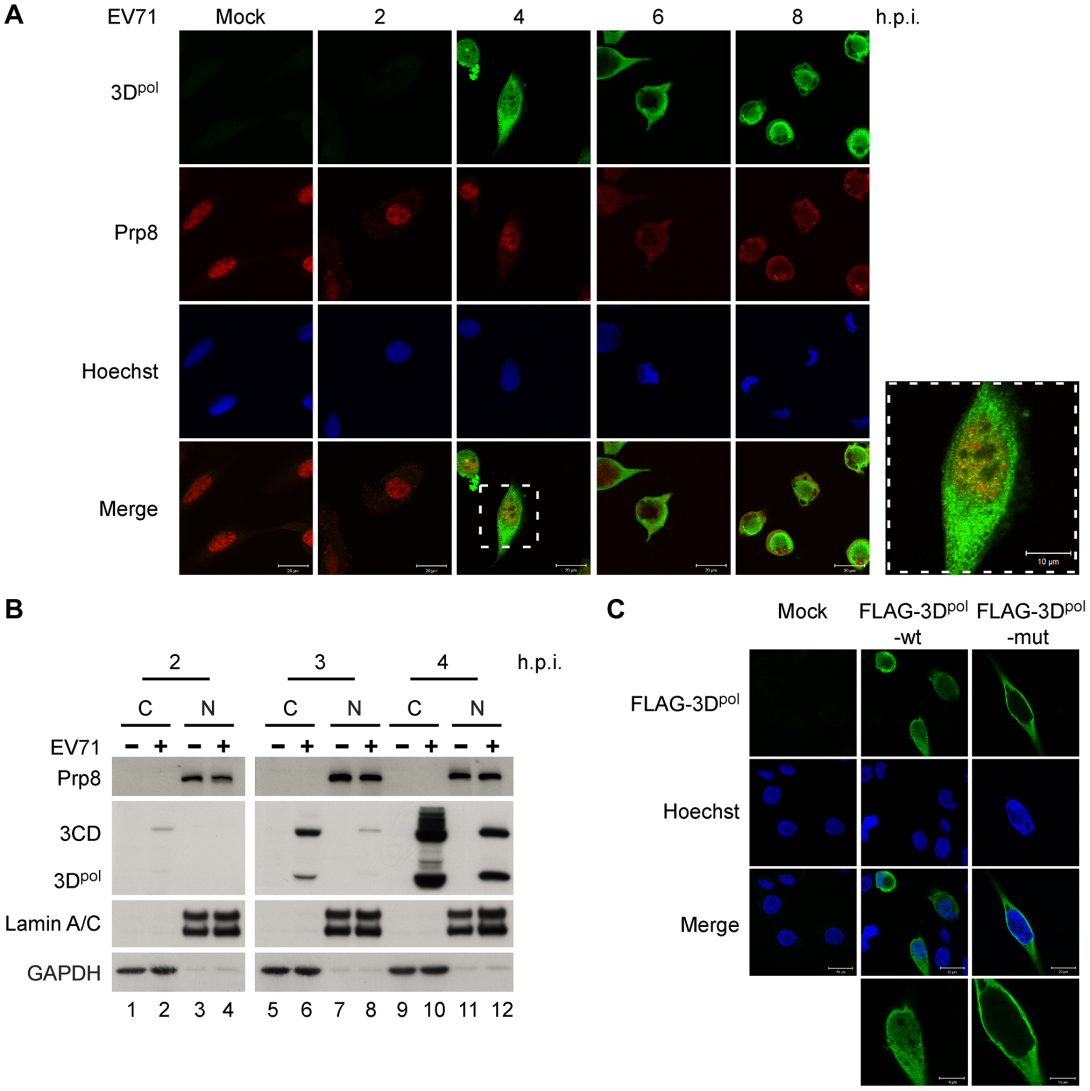
3D^pol^ and Prp8 are colocalized in the nucleus at 4 h.p.i. (A) The 3D^pol^-Prp8 association is localized in the nucleus at 4 h.p.i. Mock- or EV71 40 MOI-infected RD cells were fixed and stained using antibodies against EV71 3D^pol^ (green color) and Prp8 (red color) at 2, 4, 6, and 8 h.p.i. The nuclei of RD cells were stained with Hoechst 33258 dye (blue color), and the merged images show the 3D^pol^ and Prp8 immunofluorescence signals. All immunofluorescence images were detected by confocal microscopy. Scale bar, 10 and 20 µm. (B) 3CD, 3D^pol^, and Prp8 appear in the nuclei of infected cells at 4 h.p.i. The cytoplasmic (C) and nuclear (N) fractions of EV71-infected RD cells at 2 to 4 h.p.i were extracted and loaded with the same percent-volume for SDS-PAGE. EV71 3CD, 3D^pol^, and Prp8 were detected using anti-3D^pol^ and Prp8 antibodies in a WB assay. GAPDH and Lamin A/C were detected as cytoplasmic and nuclear protein controls, respectively. (C) EV71 3D^pol^ enters the nucleus through the KKKD amino acids of the NLS. FLAG-tagged constructs of 3D^pol^ containing the FLAG-tagged wt 126–129 aa NLS (KKKD) and mutant NLS (AAAA) were used to map the NLS on EV71 3D^pol^. RD cells were transfected with these plasmids expressing FLAG-3D^pol^-wt or FLAG-3D^pol^-mut for 48 h and then stained using antibodies against FLAG (green color). The nuclei were stained with Hoechst 33258 dye (blue color). The immunofluorescence was visualized by confocal microscopy. Scale bar, 10 and 20 µm.

The localization of 3D^pol^ and Prp8 in RD cells following a time-course of EV71 infection was studied using anti-3D^pol^ (green color) and anti-Prp8 (red color) antibodies in an immunofluorescence assay (IFA) by confocal microscopy. The images revealed that EV71 3D^pol^ was localized primarily in the cytoplasm; however, this polymerase partially entered the nucleus and was colocalized with Prp8 at 4 h.p.i. During the late stage, from 6 to 8 h.p.i., both 3D^pol^ and Prp8 were mainly present in the cytoplasm (Figure 2A). We also examined the distribution of 3D^pol^ and Prp8 in the cytoplasmic (C) and nuclear (N) fractions of EV71-infected RD cells at 2, 3, and 4 h.p.i. using the same volume percent of the cytoplasmic and nuclear extracts for WB analysis. Prp8, 3CD, and 3D^pol^ were present in the nucleus of the RD cells at 4 h post-EV71-infection (Figure 2B, lane 12). To determine whether the NLS present within the 3D^pol^ sequence impacts the nuclear entry of EV71 3D^pol^ and 3CD, the wild-type (wt) NLS sequence containing aa 126–129 (KKKD) was mutated to AAAA. Then, RD cells were transfected with FLAG-tagged 3D^pol^ with a wt or mutated NLS, and the resulting fluorescence was detected with an anti-FLAG antibody (green color) by IFA and confocal microscopy. As shown in Figure 2C, the overexpressed FLAG-3D^pol^ with the wt NLS was partially expressed in the nucleus, whereas the FLAG-3D^pol^ with the mutant NLS was only expressed in the cytoplasm of RD cells. The result indicated NLS mutation interferes with nuclear entry of FLAG-3D^pol^.

### 3D^pol^ interferes with the splicing process and promotes accumulation of the lariat form and inhibition of mRNA synthesis

**Figure 3:**
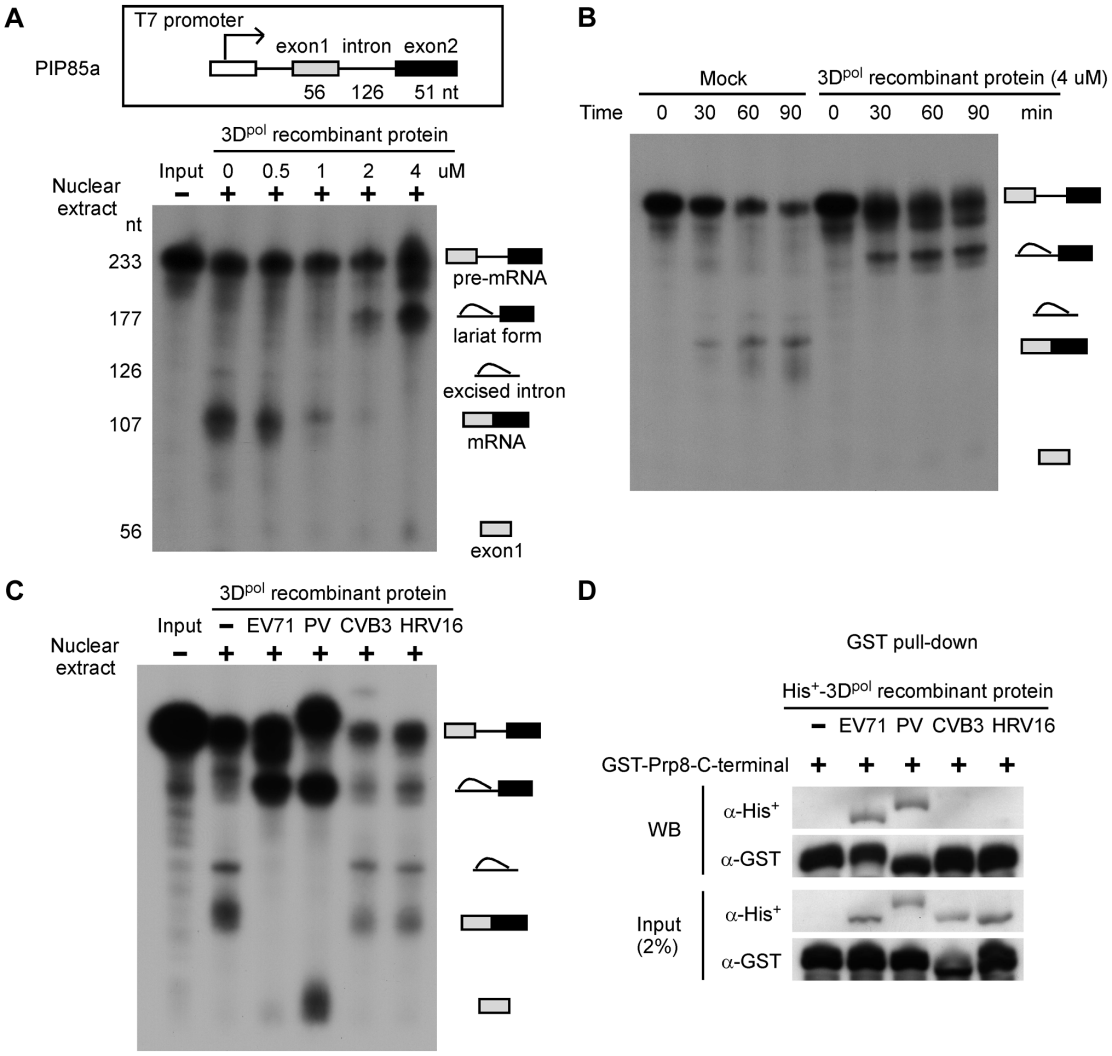
The EV71 and PV 3D^pol^ interfere with the splicing process and inhibit mRNA synthesis. (A) Recombinant EV71 3D^pol^ inhibits mature mRNA production. An *in vitro* splicing assay was performed for 90 min using ^32^P-labeled PIP85a pre-mRNA as the substrate, nuclear extracts of HeLa cells, and varying amounts of purified recombinant 3D^pol^. The autoradiogram revealed the presence of different radioactive RNA forms, including pre-mRNA, the lariat form, excised intron, mature mRNA, and exon1. (B) Recombinant EV71 3D^pol^ stops the splicing process in the lariat form. The *in vitro* splicing substrate, ^32^P-labeled PIP85a pre-mRNA, was incubated with mock- or EV71 3D^pol^ recombinant protein-containing nuclear extracts for varying time periods. The autoradiogram shows the different forms of RNAs in the splicing reaction. (C) Recombinant EV71 and PV 3D^pol^ inhibit the synthesis of mature mRNA. The *in vitro* splicing assay was performed using the same conditions described above, including a protein concentration of 4 µM and a reaction time of 90 min, with recombinant 3D^pol^ proteins from EV71, PV, CVB3, and HRV16. (D) The EV71 and PV 3D^pol^ proteins directly associate with the C-terminal domain of Prp8. *In vitro* pull-down assay, a total of 5 µg of bacterially purified His**^+^**-3D^pol^ from EV71, PV, CVB3, or HRV16 was mixed with 5 µg of the GST-Prp8-C-terminal domain fusion protein for 90-min reaction time, followed by GST pull-down and WB assays.

Whether picornaviral polymerase play a role in host pre-mRNA splicing remains unknown. Therefore, we first investigated whether 3D^pol^ affects the splicing process using *in vitro* splicing assays. PIP85a pre-mRNA was synthesized and labeled with ^32^P as the substrate, and then mixed with nuclear extracts of HeLa cells and 0.5, 1, 2, or 4 µM of EV71 3D^pol^ recombinant protein for 90 min. The splicing intermediates and products were analyzed by electrophoresis on urea-PAGE gels. The 3D^pol^ inhibited mRNA production and induced the accumulation of the lariat form in the nuclear extract, depending on the amount of EV71 3D^pol^ recombinant protein (Figure 3A). Moreover, *in vitro* splicing was evaluated using a mock treatment or treatment with 4 µM of EV71 3D^pol^ recombinant protein in a time-course study. The result showed that EV71 3D^pol^ suppressed the splicing process and produced the lariat form as early as 30 min after the start of the reaction (Figure 3B). We also determined the inhibitory splicing effect of recombinant 3D^pol^ proteins from other picornaviruses, including poliovirus (PV), coxsackievirus B3 (CVB3), and human rhinovirus type 16 (HRV16), in which a NLS was identified. Following the same conditions used in the *in vitro* splicing analysis, we discovered that PV 3D^pol^ inhibited the splicing process and led to a decrease in mRNA production and accumulation of the lariat form, similar to the results for EV71; however, neither the CVB3 nor the HRV16 3D^pol^ inhibited the pre-mRNA splicing process (Figure 3C). These data demonstrate that the 3D^pol^ of EV71 and PV blocked the second catalytic splicing step involving 3′SS cleavage and exon ligation, leading to the accumulation of the lariat form and a decrease on mRNA levels. Because Prp8 is involved in the second catalytic step of pre-mRNA splicing, we next assessed whether the inhibitory effects of these picornaviruses on the splicing process were related to Prp8. Purified His**^+^**-3D^pol^ from various picornaviruses and GST-Prp8-C-terminal region fusion proteins were mixed and subjected to GST pull-down and WB assays. The results of these assays revealed that the 3D^pol^ proteins of EV71 and PV directly associate with the C-terminal region of Prp8, whereas the CVB3 and HRV16 3D^pol^ do not (Figure 3D). Therefore, the 3D^pol^-Prp8 interaction is required for inhibition of the second catalytic step. These results suggest that the 3D^pol^ of EV71 and PV are associated with the splicing factor Prp8 and affect the normal function of Prp8 during the second catalytic splicing step, leading to inhibition of pre-mRNA splicing, accumulation of the lariat form, and a decrease in the resulting mRNA.

### 3D^pol^ inhibits intracellular pre-mRNA splicing by interacting with Prp8

**Figure 4:**
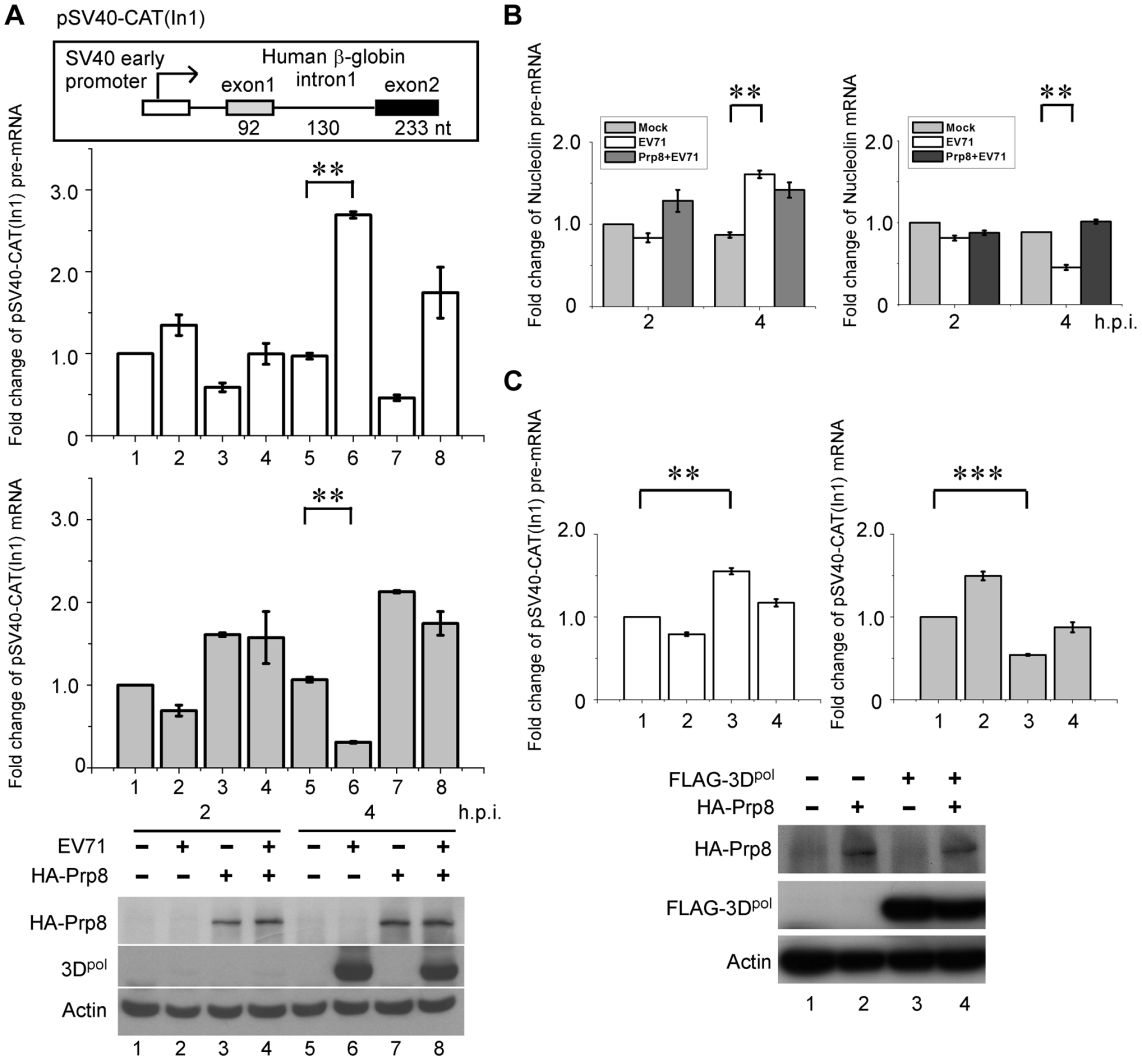
3D^pol^ affects cellular pre-mRNA splicing by interacting with Prp8. (A) EV71 inhibits cellular pre-mRNA splicing by interacting with Prp8. RD cells were transfected with pCMV-HA (lanes 1, 2, 5, and 6) or the vector encoding HA-tagged Prp8 (lanes 3, 4, 7, and 8). After 24 h, the exogenous reporter pSV40-CAT(In1), which encodes chloramphenicol acetyl transferase inserted by human β-globin intron 1, was transfected into all of the samples for 24 h. The total RNA obtained from RD cells was subsequently harvested after EV71 40 MOI infection at 2 h.p.i. (lanes 2 and 4) and 4 h.p.i. (lanes 6 and 8) for RT-qPCR. The fold changes in the amount of pre-mRNA and mRNA were calculated. The overexpression of HA-tagged Prp8 and the level of viral 3D^pol^ in infected cells were detected using anti-HA and anti-EV71 3D^pol^ antibodies, respectively, in a WB assay. (B) To confirm the effects of EV71 on endogenous splicing, RNAs were isolated from EV71-infected cells at 2 to 4 h.p.i. and evaluated with a specific primer for nucleolin by RT-qPCR. (C) 3D^pol^ inhibits cellular pre-mRNA splicing by interacting with Prp8. RD cells were transfected with constructs encoding FLAG-tagged 3D^pol^ (lanes 3 and 4) or HA-tagged Prp8 (lanes 2 and 4). The vectors pFLAG-CMV2 and pCMV-HA were used as negative controls (lane 1). The exogenous reporter pSV40-CAT(In1) was transfected into all of the samples for 24 h, and the total RNA obtained was subsequently harvested from RD cells for RT-qPCR. The fold changes in the amount of pre-mRNA and mRNA were calculated. In a WB assay, the overexpression of HA-tagged Prp8 and the level of FLAG-tagged 3D^pol^ were detected using anti-HA and anti-FLAG antibodies, respectively. Error bars, mean ± SD (n = 3). The statistical significance was analyzed using a *t* test. ^***^p<0.001; ^**^p<0.01.

We next sought to assess the effects of pre-mRNA splicing upon picornaviral infection. First, we investigated whether EV71 affects the splicing process by interfering the core splicing factor Prp8. The splicing reporter pSV40-CAT(In1) containing human β-globin intron 1 [43], [44], which encodes chloramphenicol acetyl transferase, was transfected to RD cells. After viral infection for 2 and 4 h, the RNA expression of the reporter plasmid was measured by reverse transcription quantitative PCR (RT-qPCR). EV71 infection significantly inhibited the splicing of the reporter transcription at 4 h.p.i., resulting an accumulation of the pre-mRNA and a reduction of the mRNA (Figure 4A, lane 5 vs. 6). Consistently, the splicing activity of EV71-infected cells could be restored by the overexpression of HA-tagged Prp8 (Figure 4A, lane 6 vs. 8). To confirm whether EV71 interferes with the endogenous gene splicing process, we designed specific primers for the precursor and mature RNA of the endogenous *NCL* gene, which encodes the protein nucleolin. The RT-qPCR data revealed that EV71 could suppress endogenous nucleolin pre-mRNA splicing by reacting with Prp8 (Figure 4B), similar to the results obtained by using the exogenous reporter described above. To further confirm that EV71 inhibits the pre-mRNA splicing process through the interaction of viral 3D^pol^ and host protein Prp8, the pSV40-CAT(In1) reporter was co-transfected with FLAG-tagged 3D^pol^ and HA-tagged Prp8 to RD cells. 3D^pol^ alone inhibited the splicing process, and led to increased levels of pre-mRNA and decreased levels of mRNA (Figure 4C, lane 1 vs. 3), whereas overexpression of both 3D^pol^ and Prp8 restored the pre-mRNA splicing activity (Figure 4C, lane 3 vs. 4). These results demonstrate that EV71 affects the cellular splicing process through the interaction between 3D^pol^ and Prp8 at 4 h.p.i.

### High-throughput sequence screening of the target pre-mRNA captured by Prp8 associated with 3D^pol^

**Figure 5:**
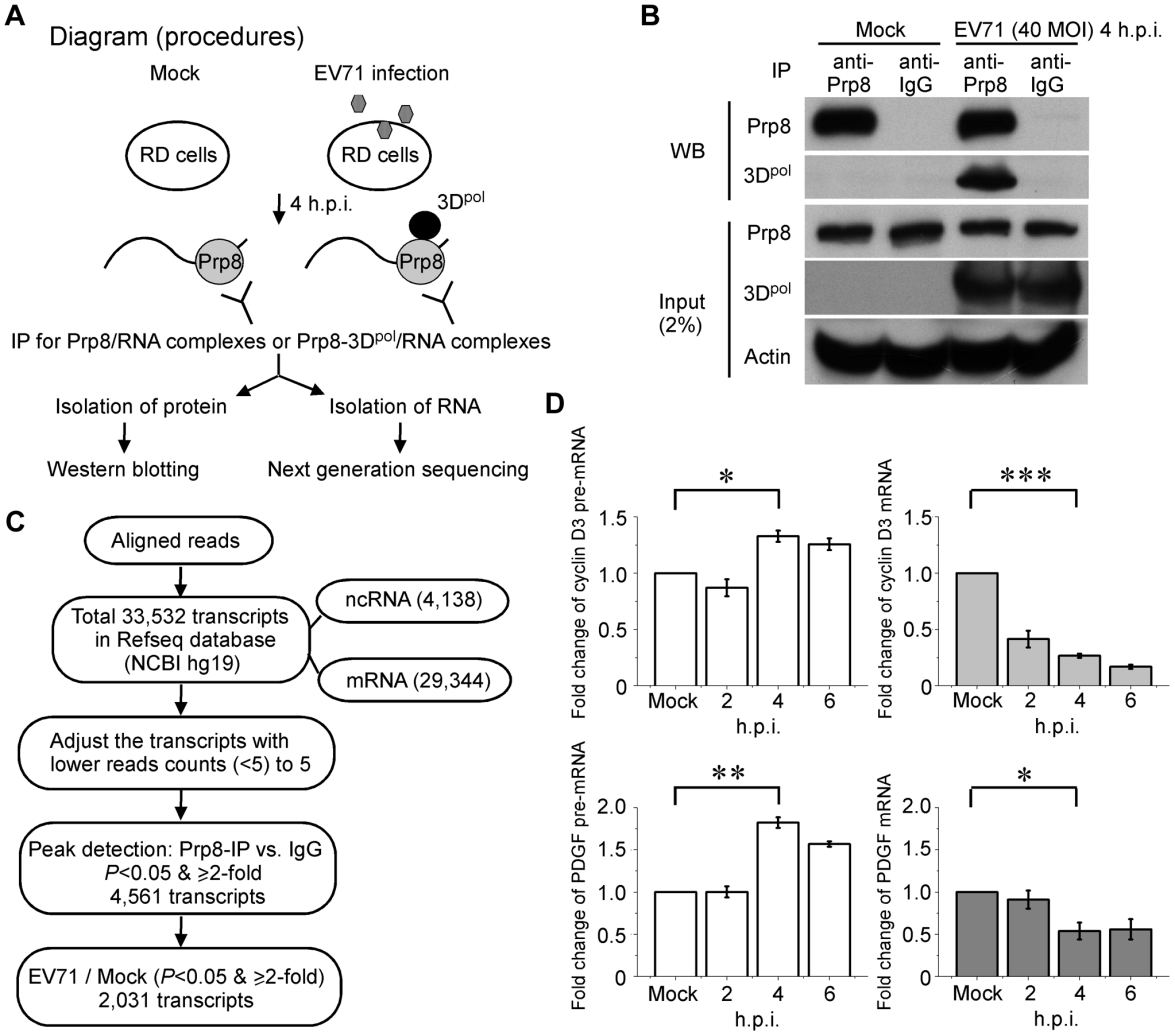
RIP-seq of the pre-mRNA trapped by the Prp8-3D^pol^ complexes. (A) Procedural diagram for the RIP assays. The schematic shows the experimental procedure for characterizing RNA from Prp8 or Prp8-3D^pol^ complexes by IP. (B) The Prp8 antibody pulls down the Prp8 and Prp8-3D^pol^ complexes. In the WB analysis, the pulled down Prp8 and Prp8-3D^pol^ complexes demonstrated the efficiency of Prp8 IP and the interaction between Prp8 and 3D^pol^. (C) A flow chart for the selection of targeted RNAs from the sequencing data. The schematic shows the experimental procedure for characterizing RNAs from Prp8 or Prp8-3D^pol^ complexes. (D) The inhibition of the pre-mRNA splicing in intracellular targeted cyclin D3 and PDGF. The increase in pre-mRNA and decrease in mRNA for intracellular cyclin D3 and PDGF in EV71-infected cells at 4–6 h.p.i. validated our RIP-Seq analysis. Error bars, mean ± SD (n = 3). The statistical significance was analyzed using a *t* test. ^***^p<0.001; ^**^p<0.01; ^*^p<0.05.

After demonstrating that 3D^pol^ associates with Prp8 and affects its function during the second step of the splicing process, leading to the accumulation of pre-mRNA intermediates (Figure 3A and 3B), we further analyzed whether endogenous pre-mRNA substrates of Prp8 associate with 3D^pol^ in EV71-infected RD cells. A diagram for the experimental procedure is provided in Figure 5A. We isolated RD cell lysates that were either infected with EV71 at a MOI of 40 for 4 h or without infection, and then a Prp8 antibody was applied to pull-down the Prp8/RNA or Prp8-3D^pol^/RNA complexes in the lysates, named RNA-binding protein IP (RIP) assay. After the Co-IP of protein-RNA complex, the proteins in the precipitates were analyzed by WB analysis. The RNAs in the precipitates were also isolated and subjected to next generation sequencing (NGS) analysis (Figure 5A). Firstly, we verified the binding of Prp8-3D^pol^ in EV71-infected cells by IP assay with the anti-Prp8 antibody. The Prp8 antibody specifically bound to Prp8, and the Prp8-3D^pol^ interaction was detected in the Prp8-3D^pol^/RNA complexes from EV71-infected cells. An antibody against IgG was used as a negative control (Figure 5B). The selection of target RNA for the NGS analysis is illustrated in the flow chart. The sequences were aligned to the human reference genome (hg19, GRCh37) and the aligned regions were annotated as known transcripts based on the Reference Sequence Database (Refseq) of the National Center of Biotechnology Information (NCBI). There are total 33,532 transcripts in Refseq database, including 29,344 mRNA and 4,138 non-coding RNA sequences. We then adjusted the transcripts with read counts lower than 5 (<5) to 5. The peaks detected for the transcripts that were pulled down by Prp8 were compared with those for IgG and were filtered for *P*<0.05 and ≥2-fold enrichment. Among the 4,561 transcripts, after filtering for *P*<0.05 and ≥2-fold, 2,031 transcripts were more highly expressed following EV71 infection than mock infection (Figure 5C). The differentially expressed transcripts were uploaded to DAVID Tools [45], and functional annotations were assigned to the KEGG pathways (Table 2). The comparison of the targeted RNA in EV71 infection and mock infection functionally associated with cell growth, proliferation, and differentiation, and these associations were related to focal adhesion and the mitogen-activated protein kinase (MAPK) pathway (Figure S1A and S1B; red aster). To validate our RIP-Seq analysis, the intracellular targeted RNAs, such as cyclin D3 of focal adhesion pathway and platelet-derived growth factor (PDGF) of MAPK pathway, were further investigated to confirm the increased levels of pre-mRNA and decreased mRNA levels at 4 to 6 h.p.i. in EV71-infected RD cells by RT-qPCR (Figure 5D).

**Table 2:** The differentially expressed transcripts were classed into groups according to functional annotations from the KEGG pathways.

Term	Count	%	PValue	List Total	Pop Hits	Pop Total
hsa04510:Focal adhesion	40	2.3474178	5.28E-05	523	201	5085
hsa04010:MAPK signaling pathway	47	2.7582160	2.34E-04	523	267	5085
hsa05200:Pathways in cancer	50	2.9342723	0.00380621	523	328	5085
hsa05221:Acute myeloid leukemia	14	0.8215962	0.00497523	523	58	5085
hsa04810:Regulation of actin cytoskeleton	35	2.0539906	0.00635485	523	215	5085
hsa05215:Prostate cancer	18	1.0563380	0.00807954	523	89	5085
hsa05220:Chronic myeloid leukemia	16	0.9389671	0.00814590	523	75	5085
hsa00030:Pentose phosphate pathway	8	0.4694836	0.01069550	523	25	5085
hsa04144:Endocytosis	30	1.7605634	0.01179329	523	184	5085
hsa04722:Neurotrophin signaling pathway	22	1.2910798	0.01396748	523	124	5085
hsa00562:Inositol phosphate metabolism	12	0.7042254	0.01921380	523	54	5085
hsa05216:Thyroid cancer	8	0.4694836	0.02424746	523	29	5085
hsa04910:Insulin signaling pathway	22	1.2910798	0.03344581	523	135	5085
hsa04540:Gap junction	16	0.9389671	0.03585054	523	89	5085
hsa04115:p53 signaling pathway	13	0.7629108	0.04190497	523	68	5085
hsa05016:Huntington's disease	27	1.5845070	0.04431270	523	180	5085
hsa05211:Renal cell carcinoma	13	0.7629108	0.05081584	523	70	5085
hsa00051:Fructose and mannose metabolism	8	0.4694836	0.05358196	523	34	5085
hsa03010:Ribosome	15	0.8802817	0.05820913	523	87	5085
hsa05219:Bladder cancer	9	0.5281690	0.06040354	523	42	5085
hsa05414:Dilated cardiomyopathy	15	0.8802817	0.08490081	523	92	5085
hsa04512:ECM-receptor interaction	14	0.8215962	0.08516606	523	84	5085
hsa04142:Lysosome	18	1.0563380	0.08763026	523	117	5085
hsa04530:Tight junction	20	1.1737089	0.08958635	523	134	5085
hsa04730:Long-term depression	12	0.7042254	0.09139957	523	69	5085
hsa04520:Adherens junction	13	0.7629108	0.09179898	523	77	5085
hsa00600:Sphingolipid metabolism	8	0.4694836	0.09875063	523	39	5085

Footnotes.

Term: gene set name.

Count: number of genes associated with this gene set.

Percentage (%): gene associated with this gene set/total number of query genes.

P-value: modified Fisher Exact P-value.

List Total: number of genes in your query list mapped to any gene set in this ontology.

Pop Hits: number of genes annotated to this gene set on the background list.

Pop Total: number of genes on the background list mapped to any gene set in this ontology.

## Discussion

**Figure 6:**
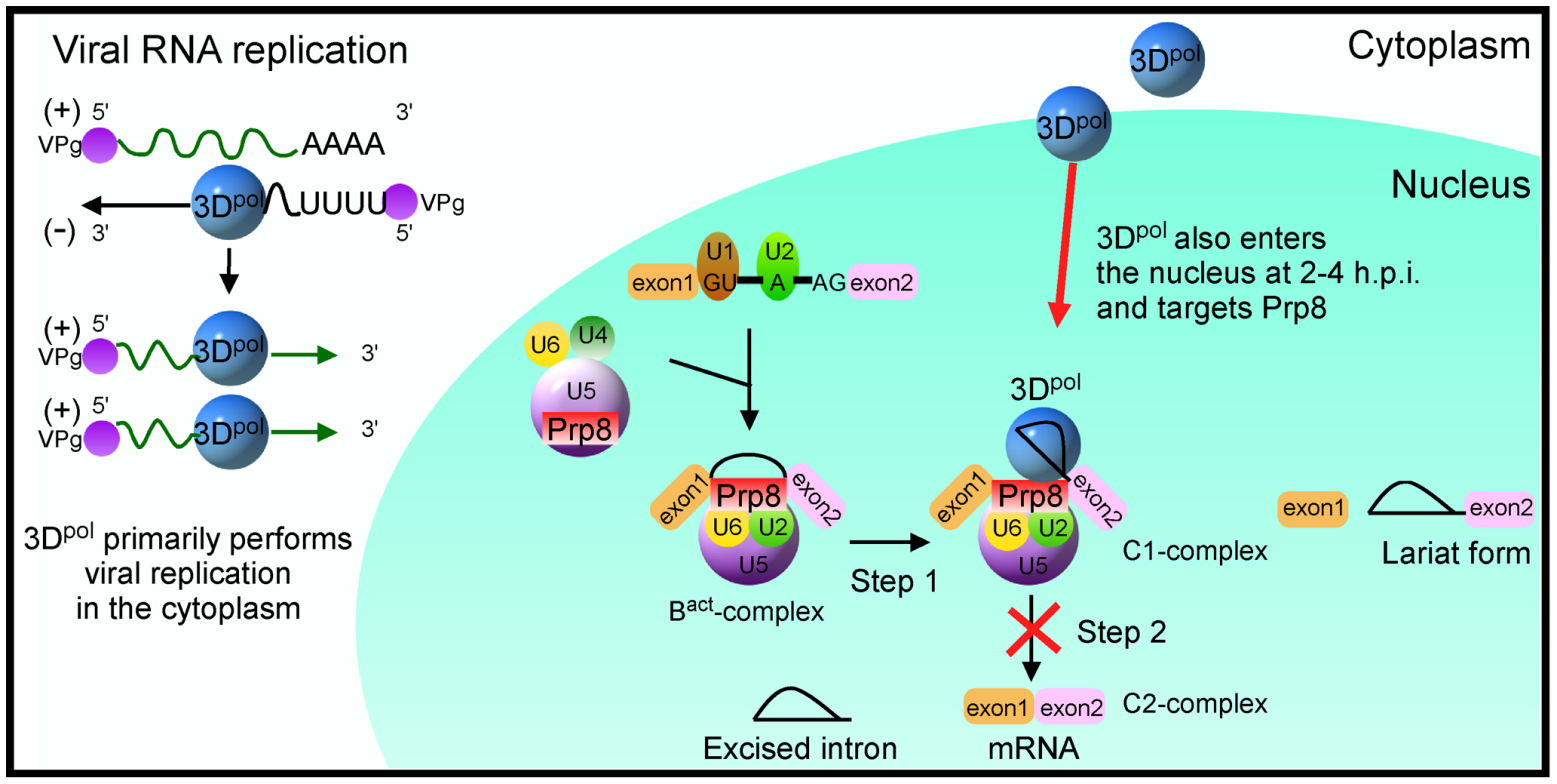
Schematic model of 3D^pol^-mediated inhibition of the cellular splicing by targeting Prp8 in the nucleus. 3D^pol^ primarily performs viral RNA replication in the host cytoplasm, but partially 3D^pol^ also enters the nucleus and interacts with the core splicing factor Prp8, which interferes with the function of Prp8 in the C1-complex. The interference of Prp8 function inhibits the second step of the splicing process and results in the accumulation of the lariat form and a reduction in mRNA synthesis.

Picornaviral 3D^pol^ plays a key role in viral genome replication in the cytoplasm. Both picornaviral 3CD and 3D^pol^ have also been observed in the nucleus as a result of infection, and this localization is mediated through the NLS of 3D^pol^. In the nucleus, mature 3C from the precursor 3CD shuts off host cell transcription [2], [19], [22]. Although evidence for the entry of picornaviral 3D^pol^ into the nucleus was first reported approximately a decade ago, the precise role of 3D^pol^ in the host nucleus has remained unknown. A schematic model is provided in Figure 6. Our study uncovered a novel mechanism for picornaviral 3D^pol^ invasion of host cells by its localization to the nucleus and association with the Prp8 protein, which is located at the center of the spliceosome. The viral 3D^pol^ affects the splicing function of Prp8 in the C1-complex and inhibits the second step of the splicing process, resulting in accumulation of the lariat form and a reduction on mRNA levels. The intracellular targeted RNAs that are trapped by the Prp8-3D^pol^ complexes are primarily responsible for cell growth, proliferation, and differentiation.

The host nuclear protein Sam68 has been shown to interact with PV 3D^pol^ using a yeast two-hybrid system, but the function of this Sam68-3D^pol^ interaction remains unknown [46]. In this study, we identified 15 novel proteins that act as host substrates for EV71 3D^pol^ using IP assays with a 3D^pol^ monoclonal antibody and MALDI-TOF MS analysis. We further selected the nuclear protein Prp8, which occupies a central position in U5 snRNP complexes, for further analysis and confirmed the interaction between endogenous Prp8 and viral 3D^pol^ without the intermediation of RNA. Prp8 provides a large platform for the RNA helicase Brr2, the GTPase Snu114, and Prp6 to form U5 snRNP complexes [33], [37]. Our results revealed that 3D^pol^ interacts with Prp8 and could be pulled down with other components of U5 snRNPs, including Brr2, Snu114, Prp6, and SNRNP40, by Co-IP and WB analysis. We further demonstrated that the fingers domain (1–286 aa) of 3D^pol^ associates with the C-terminal region (2094–2335 aa) containing the Jab1/MPN domain of Prp8 by overexpressing various truncated forms of Prp8. Moreover, the 3D^pol^ proteins of EV71 and PV directly associate with the C-terminal region of Prp8 in *in vitro* pull-down assays.

The 3D^pol^ of picornaviruses, such as PV, EMCV, HRV16, and human parechovirus-1 (HPEV-1), can enter the nucleus upon viral infection due to the expression of a NLS [19]–[22]. This NLS is partially contained within a conserved sequence, KKRD (126–129 aa), that is present in all known picornaviral RNA polymerases and may therefore play a crucial function in the life cycle of the virus [22]. However, whether EV71 can enter the nucleus in virus-infected cells remains unknown. In this study, we first observed that EV71 3D^pol^ entered the nucleus during the early stages of viral entry at 4 h.p.i., as demonstrated by anti-3D^pol^ antibody detection by confocal imaging and nuclear fractionation analysis. In contrast to PV, 3D^pol^ alone could directly and independently enter the nucleus without EV71 infection via the NLS KKKD, which spans aa 126–129. However, mutation of the NLS of 3D^pol^ by replacing the sequence KKKD with AAAA prevented nuclear entry. We also observed that 3D^pol^ and Prp8 were colocalized at 4 h.p.i. in the nucleus and at 6–8 h.p.i. in the cytoplasm of EV71-infected cells. Furthermore, the 3D^pol^-Prp8 interaction was maintained between 4 and 8 h.p.i. These results suggest that this interaction blocks the cellular pre-mRNA splicing process at the early stages of viral entry and may have advantages for the viral life cycle during the later stages. Picornavirus infection inhibits the cellular translation machinery of the host, reducing the accumulation of the cellular proteins, including Prp8 at 8 h.p.i. (Figure 1C). Furthermore, 3D^pol^ and Prp8 were co-localized in the nucleus at 4 h.p.i. and in the cytoplasm at 8 h.p.i. (Figure 2A). This interaction in the nucleus disrupts the cellular splicing machinery of the host, whereas the interaction in the cytoplasm may support the function of 3D^pol^ during viral infection, and this phenomenon is of worthy of further exploration.

Previous studies have reported that picornaviruses influence host cell gene expression by shutting off cellular transcription and cap-dependent mRNA translation [2], [47]–[49]. Poliovirus 2A protease modulates the cellular alternative splicing [50]. In this study, we examined whether picornaviral polymerase could impair cellular pre-mRNA splicing processes by interfering with Prp8. The *in vitro* splicing results demonstrated that the second step of the splicing process was blocked by the 3D^pol^ of EV71 and PV, leading to inhibition of pre-mRNA splicing, the accumulation of the lariat form, and a decrease in mRNA synthesis. However, the 3D^pol^ of CVB3 and HRV16 did not inhibit pre-mRNA splicing and did not exhibit any association with the C-terminal region of Prp8. Therefore, our data support the theory that the viral 3D^pol^ inhibits pre-mRNA splicing through an association with cellular Prp8 in the nucleus. However, the splicing effect of the 3D^pol^ of EV71 and PV in the cellular nucleus differs from that of CVB3 and HRV16, which represents a promising theme for future research.

Moreover, we demonstrated that EV71 infection inhibited the splicing of exogenous pSV40-CAT(In1) and endogenous nucleolin. However, the splicing activity of the viral infected cells could be restored by overexpression of HA-tagged Prp8. We also confirmed that 3D^pol^ alone inhibits the splicing processes by reacting with cellular Prp8. Our study provides a new insight into EV71-mediated inhibition of the pre-mRNA splicing by the 3D^pol^-Prp8 interaction. We also transfected FLAG-CVB3-3D^pol^ plasmid DNA into RD cells. The results indicated that FLAG-CVB3-3D^pol^ was unable to inhibit the splicing process (lane 1 vs. 3), and did not lead to increased levels of pre-mRNA. However, FLAG-CVB3-3D^pol^ was capable of decreasing the levels of both pre-mRNA and mature mRNA (Figure S2A). This observation was also confirmed using an in vitro splicing assay (Figure 3C) that examined the CVB3 3D^pol^ recombinant protein versus a control without 3D^pol^. These findings suggest that FLAG-EV71-3D^pol^ can inhibit splicing through interference with Prp8, whereas FLAG-CVB3-3D^pol^ did not. RD cells were infected with CVB3, a non-Prp8 interacting virus, at 2 to 6 h.p.i. (Figure S2B). The data indicated that CVB3 was unable to inhibit the splicing process at 4 h.p.i., whereas it was capable of decreasing the pre-mRNA and mature mRNA of cyclin D3 and PDGF. This result is consistent with those obtained with RD cells transfected with FLAG-CVB3-3D^pol^ (Figure S2A).

Using high-throughput sequence screening of the target pre-mRNA, we evaluated in detail the types of endogenous pre-mRNAs that were captured when Prp8 was bound to 3D^pol^ in EV71-infected RD cells. Comparison of the targeted RNA in EV71- and mock-infected cells revealed that most of the gene expression and transcription generating pre-mRNAs was associated with cell growth, proliferation, and differentiation, processes classified in the focal adhesion and MAPK pathways. In addition, the 3D^pol^-Prp8 interaction complexes disrupted mRNA synthesis and downstream protein expression. Cell-matrix adhesions are critical for biological processes such as cell motility, proliferation, differentiation, regulation of gene expression, and cell survival, and the MAPK cascade is a highly conserved pathway that is involved in numerous cellular functions, such as cell proliferation, differentiation, and migration. Viral 3D^pol^-mediated blockade of the pre-mRNA splicing process results in the production of less cellular mRNA and the use of more translational machinery by viral RNA to make viral proteins efficiently. The disruption of cellular mRNA synthesis by the infecting virus may also directly cause cell damage and death. Timely cell death would facilitate the release of some picornaviruses, e.g., enterovirus 71. Furthermore, certain picornavirus genome sequences may contain fortuitous splice sequences. Erroneous recognition of such sequences by cytoplasmic splicing components would require the presence of some sort of anti-splicing defensive mechanism.

In summary, our study reports a novel invasion strategy for picornaviruses in which 3D^pol^ enters the host cell nucleus and associates with Prp8. This association leads to the inhibition of pre-mRNA splicing, accumulation of the lariat form, and a decrease in mRNA synthesis. However, the pre-mRNA and the intermediate lariat form become occupied by the 3D^pol^-Prp8 interaction during infection, thereby blocking mRNA synthesis of numerous cellular genes, including those associated with cell growth, proliferation, and differentiation.

Picornaviruses inhibit the cellular transcription and cap-dependent mRNA translation to affect the genes expression of the host cell [2], [47]–[49]. However, several cellular genes could escape the shutoff of gene expression by picornavirus infection. Our previous investigations of cDNA microarray analysis for total cellular RNA demonstrated that the level of some RNAs that are related to chemokines, protein degradation, complement proteins and proapoptosis proteins increased upon EV71 infection, suggesting leakage from the inhibition of transcription by EV71 [51]. Translations of c-myc, Bip, and eIF4G mRNA have been observed to be increased in poliovirus-infected cells as the cap-dependent translation shuts down, owing to the presence of internal ribosome entry sites (IRES) [52]–[54]. This work presents a novel mechanism by which cytoplasmic viral RdRp inhibits cellular gene expression in the pre-mRNA splicing processing step, which would inhibit mRNA synthesis of the surviving host RNAs, potentially providing yet another advantage of virus replication.

## Materials and Methods

### Cell cultures and virus infection

Human RD, HEK293T, and HeLa cells were cultured in DMEM containing FBS and penicillin/streptomycin/glutamine (Gibco) at 37°C. EV71 (TW/4643/98) virus infection at a MOI of 40 was performed under serum-free conditions for 1 h at 37°C. After 1 h of incubation, the virus-infected cells were washed twice in PBS, and the medium was replaced with DMEM containing 2% FBS to maintain the virus-infected cells at 37°C.

### Immunoprecipitation and protein identification

To identify potential EV71 3D^pol^-interacting host proteins, 5 mg of cell lysates from EV71 40 MOI-infected RD cells at 6 h.p.i. was harvested for immunoprecipitation and treated with 250 µg of EV71 3D^pol^ monoclonal antibody (self-preparation) and 100 µl of protein A-Sepharose (GE Healthcare) at 4°C. After centrifugation and bead washing, the co-precipitated proteins were separated by 8–16% gradient SDS-PAGE, which was followed by silver staining. The proteins were identified using in-gel digestion and analyzed by Bruker Ultraflex MALDI-TOF MS. Mass lists were performed peptide mass fingerprinting by Biotool 2.0 software and the algorithm of Mascot (http://www.matrixscience.com).

### Co-immunoprecipitation and western blotting

RD cells (2.4×10^6^/10-cm dish) were seeded 24 h prior to EV71 infection at a MOI of 40. Cells were lysed in 1 ml of IP-lysis buffer (25 mM Tris-HCl pH 7.6, 300 mM NaCl, 0.5% CA630, 1.5 mM MgCl_2_, 0.2 mM EDTA, 0.5 mM DTT, and 1× proteinase inhibitor) at 4°C for 30 min and then treated with 10 µg/ml RNase A at 30°C for 1 h. The cell extracts were pre-cleared by incubation at 4°C for 1 h with protein G-agarose (GE Healthcare) and centrifuged to remove non-specific complexes. The lysate was then added to 10 µg/ml EV71 3D^pol^ antibody or Prp8 antibody (Abcam) at 4°C for 2 h and 100 µl of protein G-agarose at 4°C for 12 h. The co-precipitated proteins were collected by centrifugation, followed by washing 6 times with IP buffer (25 mM Tris-HCl pH 7.6, 200 mM NaCl, 0.1% CA630, 6% glycerol, 1 mM EDTA, 0.5 mM DTT, and 1× proteinase inhibitor). The precipitated proteins were separated by 8% SDS-PAGE; subsequently, these immune complexes were detected using anti-Prp8 (diluted 1∶5000; Abcam), Brr2 (diluted 1∶5000; Abcam), Snu114 (diluted 1∶5000; Abcam), Prp6 (diluted 1∶5000; Abcam), SNRNP40 (diluted 1∶5000; Abcam), EV71 3D^pol^ (diluted 1∶10000; self-preparation), and β-actin (diluted 1∶10000; Millipore) antibodies in a WB assay.

### Plasmid construction and FLAG immunoprecipitation

To map the interacting domains between 3D^pol^ and Prp8, the full-length and various truncated forms of human Prp8 were amplified by PCR from the human Prp8-pCMV-XL5 cDNA clone (OriGene) using specific primers. The PCR product was inserted into a pCMV-HA vector (Clontech) between the XhoI and NotI sites to enable the expression of HA-tagged proteins. The EV71 full-length infection cDNA clone was used to amplify full-length and various truncated forms of EV71 3D^pol^ by PCR, followed by cloning into the EcoRI and KpnI sites of the p3XFLAG-Myc-CMV-25 vector (Sigma) to enable expression of the EV71 3D^pol^ constructs as fusions with 3 adjacent FLAG epitopes. To overexpress these proteins, 2 µg of the constructs of the various truncated forms of Prp8 and 3D^pol^ was co-transfected into HEK293T cells (1×10^6^/per 6-well plate) using Lipofectamine 2000 reagent (Invitrogen) for 48 h. The cells were harvested for FLAG-IP using a FLAG-immunoprecipitation kit (Sigma). After lysis and centrifugation, the supernatant was treated with 10 µg/ml RNase A at 30°C for 1 h, and then 40 µl of anti-FLAG M2 agarose affinity gel was added at 4°C for 12 h. Proteins were then eluted by competition with 3×FLAG peptide. In the WB assay, the precipitated proteins were identified using an anti-HA antibody (diluted 1∶5000; Sigma) and an anti-FLAG antibody (diluted 1∶5000; Sigma).

### Construction of the mutant FLAG-3D^pol^ NLS, immunofluorescence microscopic analysis, and cellular fractionation

The PCR product of EV71 3D^pol^ containing the wt NLS (KKKD) was cloned into the pFLAG-CMV-2 vector (Sigma) between the EcoRI and KpnI sites to enable expression of the protein as a fusion with the FLAG epitope protein. The mutated NLS (AAAA)-containing pFLAG-3D^pol^ clone was generated using specific primers (Table S1) in 2 steps of PCR and digestion and subsequently cloned into the pFLAG-CMV-2 vector. The wt and mutant clones of EV71 3D^pol^ were verified by sequencing. For immunofluorescence microscopic analysis, RD cells grown in 22-mm-diameter wells at 80% confluency were infected with EV71 at a MOI of 40 for 2 to 8 h.p.i. or were transfected with 4 µg of the wild-/mutant-type 3D^pol^ clone. The cells were fixed in PBS containing 4% formaldehyde, permeated with 0.3% Triton X-100, blocked with 0.5% BSA for 1 h at 25°C, and then stained with anti-EV71 3D^pol^ (diluted 1∶200; self-preparation), anti-Prp8 (diluted 1∶15; Abcam), or anti-FLAG (diluted 1∶200; Sigma) antibodies for 2 h at 37°C. Subsequently, the cells were stained with FITC-conjugated goat anti-mouse IgG (diluted 1∶400; green; Invitrogen) or goat anti-rabbit IgG (diluted 1∶75; red; Invitrogen) for 2 h at 37°C, followed by treatment with nuclear Hoechst 33258 stain (diluted 1∶500; blue) for 15 min at 25°C. The cells were washed 3 times with PBS and mounted on glass slides with Prolong Gold (Invitrogen). Confocal images were obtained with a confocal laser-scanning microscope (Zeiss; LSM 510 NLO). To prepare EV71-infected RD cells (2.4×10^6^/10-cm dish) for cytoplasmic and nuclear fractionation, the cells were lysed in 300 µl of buffer C, provided in the CMN compartment protein extraction kit (Biochain), for 30 min at 4°C and then disrupted by 50 passages through a 25G needle. After centrifugation, the pellets were washed with buffer W 3 times and lysed in 50 µl of buffer N for 1 h at 4°C. The same volume percent of cellular fractionation was loaded onto an 8% SDS-PAGE gel, and GAPDH (diluted 1∶5000; Abnova) and Lamin A/C (diluted 1∶5000; Santa Cruz) were detected as internal controls for the cytoplasmic and nuclear fractions, respectively, by WB.

### *In vivo* splicing assay and reverse transcription quantitative PCR

RD cells were transfected with 4 µg of the pCMV-HA vector or HA-tagged Prp8 clone for 24 h, followed by transfection with 0.5 µg of the pSV40-CAT(In1) splicing reporter (a gift from Dr. Woan-Yuh Tarn, Academia Sinica, Taiwan) [44] for 24 h. The RD cells were infected with EV71 at a MOI of 40 at 2 and 4 h.p.i., and RNA samples were harvested from cells using an RNeasy mini kit (Qiagen) and treated with RQ-DNase1 (Promega). RNAs were converted into first-strand cDNAs using SuperScript III reverse transcriptase (Invitrogen) with CAT(In1) reverse primers. All PCR reactions were performed using specific primers (Table S1). The qPCR analysis was performed using SYBR Green reagents and the LightCycler 480 instrument (Roche). NCBI GI numbers for genes and proteins mentioned in the text were provided in the Table S2.

### Expression and purification of Prp8-C-terminal region and 3D^pol^ recombinant proteins

The Prp8-C-terminal region (2094–2335 aa) was cloned into the EcoRI and XhoI sites of the pGEX-5X-1 vector and transformed into BL21(DE3). Expression of the protein was induced by adding 1 mM isopropyl-β-d-thiogalactopyranoside (IPTG) at 16°C for 16 h. The protein expressed from lysed cells was suspended in buffer (20 mM HEPES pH 7.9, 300 mM NaCl, 5 mM DTT, 0.2 mM EDTA, 0.05% NP-40, 0.5 mM PMSF) at 4°C for 30 min and loaded onto a GST column (GE Healthcare), which was then eluted with buffer containing 10 mM Glutathion. The eluted product was dialyzed in buffer (20 mM HEPES pH 7.9, 100 mM NaCl, 1 mM DTT, 0.2 mM EDTA, 0.01% NP-40, 20% glycerol). To construct pET26b-Ub-EV71-3D-6H, the EV71 3D^pol^ full-length containing the 6×His**^+^** at C-terminal region was cloned into the SacII and BamHI sites of pET26b-Ub-3D-GSSG-6H [55] and used to replace the PV 3D-GSSG-6H of pET26b-Ub-3D-GSSG-6H. pCG1 encodes an Ub-specific carboxy-terminal protease (Ubp1). Expression of Ub-3D fusion protein in the presence of Ubp1 has glycine at the amino terminus of polymerase, not methionine. Plasmids pET26b-Ub-EV71-3D-6H and pCG1 or pET26b-Ub-3D-GSSG-6H and pCG1 were cotransformed into BL21(DE3) to express EV71-3D-6H or PV-3D-GSSH-6H recombinant protein, respectively. Expression of these recombinant proteins were induced by adding 50 µM IPTG at 25°C for 4 h. The protein expressed from lysed cells was suspended in buffer A (50 mM Tris pH 8.0, 20% glycerol, 1 mM DTT, 0.1% NP-40, and 60 µM ZnCl_2_) and loaded onto a HisTrap column (GE Healthcare), which was then washed with buffer A containing 30, 50, 70, or 90 mM imidazole; the protein was then eluted with buffer A containing 500 mM imidazole. The eluted product was dialyzed in buffer B (50 mM HEPES, pH 7.5, 500 mM NaCl, 20% glycerol, 1 mM DTT, 0.1% NP-40, and 60 µM ZnCl_2_) [18]. The purified 3D^pol^ recombinant proteins of CVB3 and HRV16 were gifts from Dr. Craig E. Cameron. The expression and purification of CVB3 and HRV16 3D^pol^ have been described previously [55]–[57].

### *In vitro* splicing assay, and glutathione S-transferase (GST) pull-down

The PIP85a plasmid (a gift from Dr. Woan-Yuh Tarn, Academia Sinica, Taiwan) was cleaved using Hind III and labeled with α-^32^P-UTP (800 Ci/mmol) using an *in vitro* transcription system (Promega). *In vitro* splicing was performed in 15 µl of reaction mixtures containing 60% HeLa cell nuclear extracts, 10 mM ATP, 0.4 M creatine phosphate, 48 mM MgCl_2_, 12 U RNasin, various recombinant proteins, and 3×10^5^ cpm ^32^P-labeled PIP85a pre-mRNA as the substrate for 1.5 h at 37°C. RNA was extracted with the Trizol reagent (Invitrogen) from the splicing reaction and fractionated on a 5% denaturing polyacrylamide gel containing 7 M urea, TBE, APS, and TEME. A total of 5 µg of each recombinant protein was incubated in IP lysis buffer (25 mM Tris-HCl pH 7.6, 300 mM NaCl, 0.5% CA630, 1.5 mM MgCl_2_, 0.2 mM EDTA, 0.5 mM DTT, and 1× proteinase inhibitor) at 4°C for 3 h, and the targeted proteins were immunoprecipitated using GST beads and washed with IP buffer (25 mM Tris-HCl pH 7.6, 200 mM NaCl, 0.1% CA630, 6% glycerol, 1 mM EDTA, 0.5 mM DTT, and 1× proteinase inhibitor). The same concentration (5 µg) of GST-Prp8-C-terminal region (2094–2335 aa) and 6×His**^+^** viral 3D^pol^ recombinant proteins were incubated in IP-lysis buffer for 3 h at 4°C, and Glutathione Sepharose (GE Healthcare) was then added to the mixture. After denaturation in 6× loading dye and centrifugation, the precipitated proteins were separated by 10% SDS-PAGE and analyzed by WB with an anti-GST antibody (diluted 1∶5000; Santa Cruz) and an anti-His**^+^** antibody (diluted 1∶5000; Calbiochem).

### RNA-binding protein immunoprecipitation-sequencing (RIP-Seq)

The 20 dishes of Mock or EV71-infected RD cells (9.6×10^6^/15-cm dish) were harvested in IP-lysis buffer, and the lysates were then added to the Prp8 antibody or control IgG antibody (Abcam) for RIP analysis. RNA-protein complexes were immunoprecipitated with protein G agarose beads, and total RNA was extracted by treatment with proteinase K and Trizol and prepared for sequencing. RNA sequencing was performed by the Genomics Core Laboratory of the Molecular Medicine Research Center, Chang Gung University. SOLiD sequencing libraries were prepared using the SOLiD Total RNA Sequencing kit (ABI PN4445374) according to the manufacturer's instructions. Approximately 1 µg of total immunoprecipitated RNA was used as the starting material. The samples were then subjected to ribosomal RNA removal using Ribo-Zero Gold kits (human/mouse/rat) (Epicentre); subsequently, 100 ng of the rRNA-depleted RNAs was fragmented using RNase III. After purification, 50 ng of each fragmented sample was ligated with RNA adaptors. An Agilent 2100 Bioanalyzer was used to profile the distribution of the fragmented RNA (the median size was between 125 to 140 nt). After reverse transcription and size selection, each cDNA library was amplified using distinct barcoded 3′ PCR primers from the SOLiD RNA barcoding kits (PN 4427046). The insert size distribution and the concentration of each library were measured using the Agilent 2100 Bioanalyzer. From each library, equal concentrations (0.8 pM) were pooled together and sequenced strand-specifically on an ABI SOLiD5500 platform (Life Technologies, Foster City, CA, USA) to generate 75-bp tags. The single-end sequence data were aligned to the hg19 (GRCh37) human reference genome. The transcripts were uploaded to DAVID Tools, and functional annotations were assigned based on the KEGG pathways.

## Supporting Information

Figure S1 **Pathway maps.** (A) KEGG pathway entry (hsa04510) for focal adhesion. (B) KEGG pathway entry (hsa04010) for MAPK signaling pathway.
(TIF)

Figure S2 **CVB3 3D^pol^ is unable to inhibit the cellular splicing process.** (A) CVB3 3D^pol^ leads to decreased levels of both pre-mRNA and mature mRNA. RD cells were transfected with constructs encoding FLAG-tagged CVB3 3D^pol^ (lanes 3 and 4) or HA-tagged Prp8 (lanes 2 and 4). The vectors pFLAG-CMV2 and pCMV-HA were used as negative controls (lane 1). The exogenous reporter pSV40-CAT(In1) was transfected into all of the samples for 24 h, and the total RNA obtained was subsequently harvested from RD cells for RT-qPCR. The fold changes in the amount of pre-mRNA and mRNA were calculated. In a WB assay, the overexpression of HA-tagged Prp8 and the level of FLAG-tagged CVB3 3D^pol^ were detected using anti-HA and anti-FLAG antibodies, respectively. (B) CVB3 is unable to inhibit the splicing process in intracellular cyclin D3 and PDGF. CVB3 decreased the pre-mRNA and mRNA of intracellular cyclin D3 and PDGF in CVB3 40 MOI-infected RD cells.
(TIF)

Table S1 **The sequences of primers.**
(DOC)

Table S2 **A list of NCBI GI numbers for genes and proteins mentioned in the text.**
(DOC)
