# Supplementary figures and images for: Cytoplasmic Viral RNA-Dependent RNA Polymerase Disrupts the Intracellular Splicing Machinery by Entering the Nucleus and Interfering with Prp8
Source: PLoS Pathog. 2014 Jun 26;10(6):e1004199. doi: 10.1371/journal.ppat.1004199 (PMC4072778; doi:10.1371/journal.ppat.1004199)

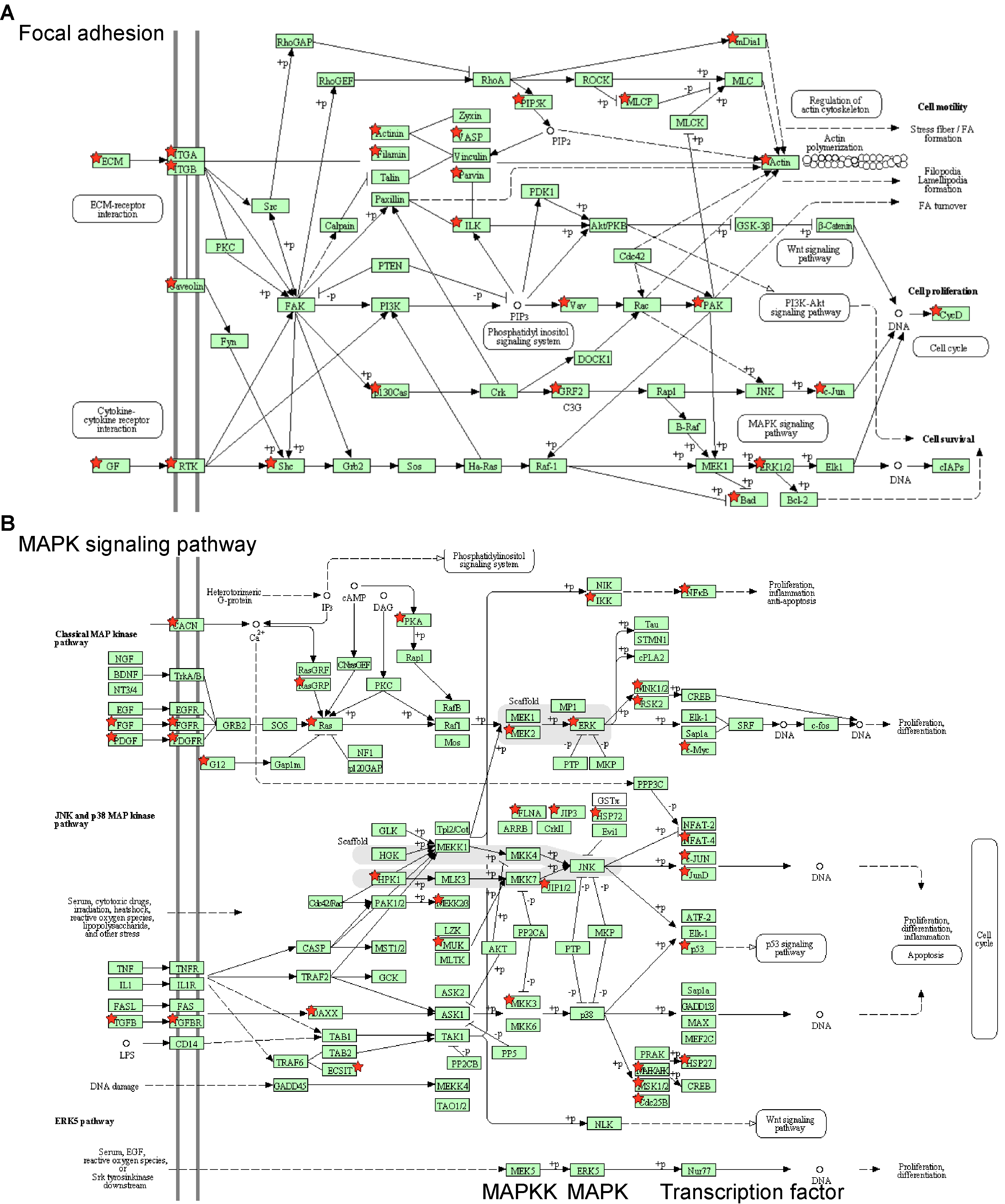

Supplement: Figure S1 — Pathway maps. (A) KEGG pathway entry (hsa04510) for focal adhesion. (B) KEGG pathway entry (hsa04010) for MAPK signaling pathway. (TIF) [file ppat.1004199.s001.tif]

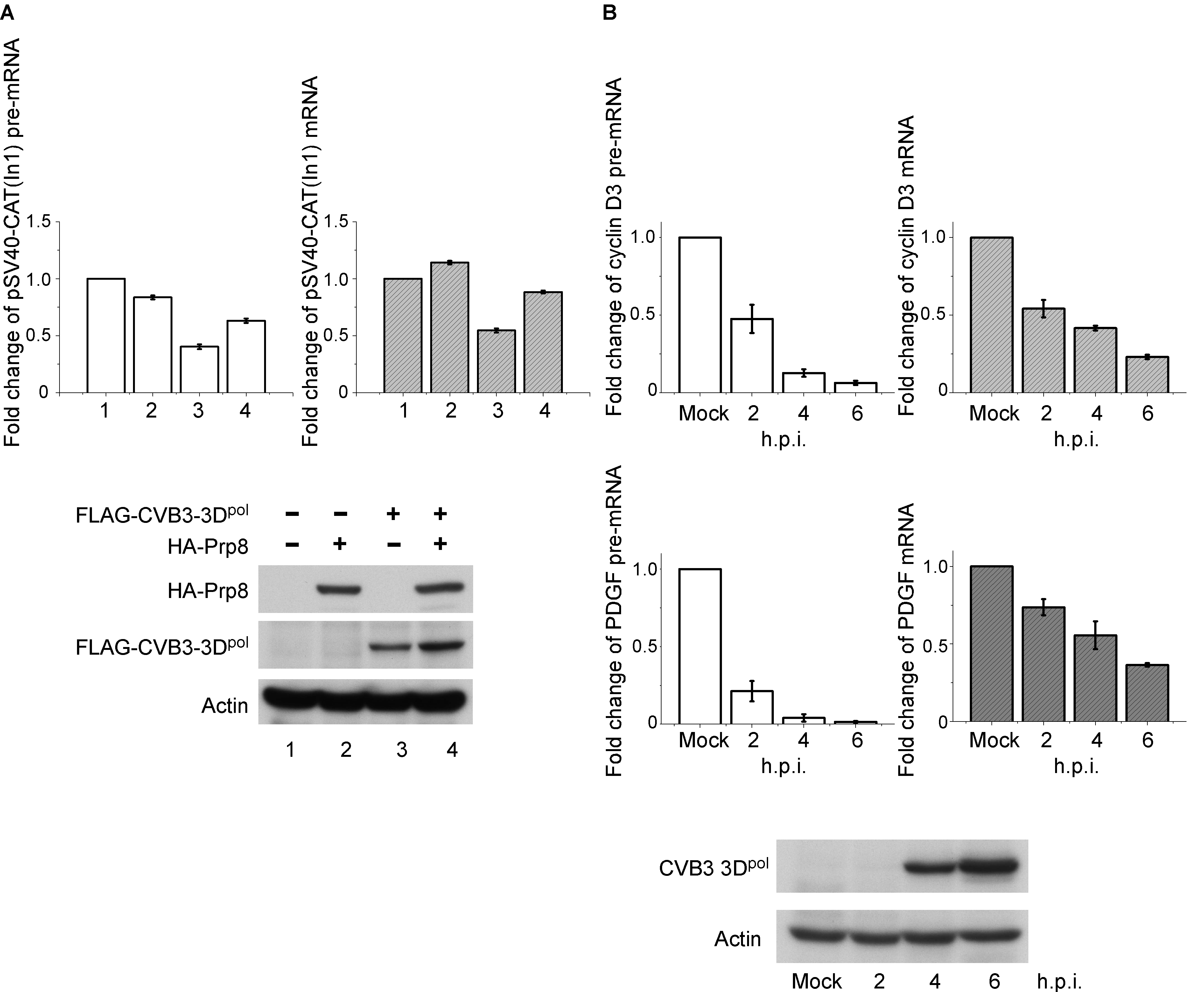

Supplement: Figure S2 — CVB3 3Dpol is unable to inhibit the cellular splicing process. (A) CVB3 3Dpol leads to decreased levels of both pre-mRNA and mature mRNA. RD cells were transfected with constructs encoding FLAG-tagged CVB3 3Dpol (lanes 3 and 4) or HA-tagged Prp8 (lanes 2 and 4). The vectors pFLAG-CMV2 and pCMV-HA were used as negative controls (lane 1). The exogenous reporter pSV40-CAT(In1) was transfected into all of the samples for 24 h, and the total RNA obtained was subsequently harvested from RD cells for RT-qPCR. The fold changes in the amount of pre-mRNA and mRNA were calculated. In a WB assay, the overexpression of HA-tagged Prp8 and the level of FLAG-tagged CVB3 3Dpol were detected using anti-HA and anti-FLAG antibodies, respectively. (B) CVB3 is unable to inhibit the splicing process in intracellular cyclin D3 and PDGF. CVB3 decreased the pre-mRNA and mRNA of intracellular cyclin D3 and PDGF in CVB3 40 MOI-infected RD cells. (TIF) [file ppat.1004199.s002.tif]
