# Supplementary material for: Cytoplasmic Viral RNA-Dependent RNA Polymerase Disrupts the Intracellular Splicing Machinery by Entering the Nucleus and Interfering with Prp8
Source: PLoS Pathog. 2014 Jun 26;10(6):e1004199. doi: 10.1371/journal.ppat.1004199 (PMC4072778; doi:10.1371/journal.ppat.1004199)
Supplement: Table S1 — The sequences of primers. (DOC) [file ppat.1004199.s003.doc]

**Table S1.** The sequences of primers

| Name | Sequence |
| --- | --- |
| pFLAG-3D^pol^-For | 5'-CCGGAATTCAGGTGAGATCCAATGGATGAAG-3' |
| pFLAG-3D^pol^-Rev | 5'-CGGGGTACCTCAAAACAATTCGAGCCAATTTCT-3' |
| pFLAG-3D^pol^-mutagenic | 5'-CGAGTTGTTGGATCCAAAATGGCCGCTGCCGCGATG |
|  | CCTAGTGCACTGTATG-3' (mutated nucleotides are underlined) |
| pSV40-CAT(In1)-exon-For | 5'-CCAGACCGTTCAGCTGGATATT -3' |
| pSV40-CAT(In1)-intron-For | 5'-ATTGGTCTATTTTCCCACCCTTAG -3' |
| pSV40-CAT(In1)-Rev | 5'-GTATTCACTCCAGAGCGATG-3' |
| nucleolin-intron-For | 5'-AATTTTTAGGTGACTTCTCTT-3' |
| nucleolin-exon-For | 5'-GACGATGAGGAAGATGACTCTGAAGA-3' |
| nucleolin-exon-Rev | 5'-TCATCCTCAGCCACGTTCT-3' |
| cyclin D3-intron-For | 5'-ACTCTTCTCCCATGTTCCCAGG-3' |
| cyclin D3-exon-For | 5'-TGGCACTGAAGTGGACTGCCT-3' |
| cyclin D3-exon-Rev | 5'-TGTAGGAGTGCTGGTCTGGC-3' |
| PDGF-intron-For | 5'-GATTGGGTTAAATGTTTTCACCCTG-3' |
| PDGF-exon-For | 5'-GCTTCCTGTCTCGCCTTTTCCT-3' |
| PDGF-exon-Rev | 5'-ACACGCCATGTACATCCATGTC-3' |

Footnotes: EV71 3D^pol^ containing the mutant NLS (AAAA) was amplified by PCR using specific primers. The sequences of primers were used for RT-qPCR.
