# Supplementary material for: Cytoplasmic Viral RNA-Dependent RNA Polymerase Disrupts the Intracellular Splicing Machinery by Entering the Nucleus and Interfering with Prp8
Source: PLoS Pathog. 2014 Jun 26;10(6):e1004199. doi: 10.1371/journal.ppat.1004199 (PMC4072778; doi:10.1371/journal.ppat.1004199)
Supplement: Table S2 — A list of NCBI GI numbers for genes and proteins mentioned in the text. (DOC) [file ppat.1004199.s004.doc]

**Table S2.** A list of NCBI GI numbers for genes and proteins mentioned in the text

| Name | NCBI GI No. |
| --- | --- |
| Pre-mRNA processing factor 8 homolog (Prp8) | GI: 39963074 |
| 200 kDa U5 snRNP-specific spliceosomal protein (Brr2) | GI: 45861372 |
| U5-116KD (Snu114) | GI: 48145665 |
| U5 snRNP associated 102 kDa protein (Prp6) | GI: 119595584 |
| U5 snRNP-specific 40 kDa protein (SNRNP40) | GI: 3820594 |
| Enterovirus 71 strain Tainan/4643/98 | GI: 10946422 |
| Poliovirus, complete genome | GI: 12408699 |
| Coxsackievirus B3 (CVB3) complete genome | GI: 323419 |
| Human rhinovirus type 16 polyprotein gene | GI: 409463 |
| Human nucleolin gene | GI: 189305 |
| Homo sapiens cyclin D3 (CCND3) gene | GI: 21397157 |
| Homo sapiens platelet-derived growth factor alpha polypeptide (PDGFα) | GI: 343488502 |
